# Optimising tibial extension stem selection in total knee arthroplasty: the role of digital modelling

**DOI:** 10.3389/fbioe.2025.1634172

**Published:** 2025-10-10

**Authors:** Mathieu Severyns, François Zot, Marc Gardegaront, Arnaud Germaneau, Tanguy Vendeuvre

**Affiliations:** ^1^ Université de Poitiers, Institut Pprime UPR 3346 CNRS, CHU de Poitiers, Service Chirurgie Orthopédique et Traumatologique, Poitiers, France; ^2^ Orthopaedic and Traumatology Department, Clinique Porte Océane, Les Sables d’Olonne, France; ^3^ Univ Lyon, Univ Eiffel, Univ Claude Bernard Lyon 1, LBMC UMR, Lyon, France

**Keywords:** total knee arthroplasty, extension stem, biomechanics, finite element analysis, patient specific

## Abstract

**Introduction:**

This study explores the biomechanical impact of tibial extension stems in total knee arthroplasty using finite element digital modelling. The objective is to enhance stem selection by assessing stress and strain distribution in periprosthetic bone under varied loading scenarios.

**Methods:**

Six patient-specific FE models were created, each with different stem dimensions, to evaluate how stem geometry affects implant stability and fracture risk.

**Results:**

Extension stems reduce strain under the tibial baseplate but increase stress and fracture risk in the surrounding bone, particularly at the stem tip. Larger stem diameters were linked to higher fracture risks due to increased press-fit contact.

**Conclusion:**

These findings are consistent with previous research emphasizing the importance of stem design in achieving a balance between implant stability and bone preservation. The study offers a biomechanical foundation for surgical planning, potentially improving TKA durability and functional outcomes. Incorporating these insights into clinical practice may enhance the longevity of knee replacements and overall patient quality of life.

## 1 Introduction

Total knee arthroplasty (TKA), also known as total knee replacement, is an increasingly common surgical intervention that aims to definitively treat symptoms of osteoarthritis in our patients (Kurtz et al., 2007; Huizinga et al., 2012). Although TKA survival rates are currently estimated at around 90% after 15 years (Frehill et al., 2015; Kim et al., 2021; Putman et al., 2018), there are still mechanical complications, particularly aseptic loosening, which can cause implant migration, which is the leading cause of early mechanical failure (Bae et al., 2012; Laver et al., 2024). Additionally, it has been shown that obesity may increase the occurrence of these mechanical complications (Druel et al., 2024). In the vast majority of cases, these much-feared complications result in a tricky surgical revision. These biomechanical complications seem to be mainly related to two factors: a pre-existing bone fragility and/or a suboptimal bone–implant interface in which loading is unevenly distributed across the epiphyseal surface (Cuckler, 2004).

One potential solution to compensate for issues of bone fragility is the addition of tibial extension stems. These devices are believed to improve primary mechanical stability by increasing the intramedullary anchoring surface and/or establishing a more robust contact zone in the cortical bone. In this respect, previous studies have shown that extension stems significantly contribute to a reduction in equivalent stresses in osteoporotic epiphyses (Mabry and Hanssen, 2007; Conlisk et al., 2018). However, these extension systems have their limits, in particular the risk of bone resorption triggered by the phenomenon of stress shielding.

The use of a digital model based on finite element (FE) method might help surgeons during preoperative planning by including an analysis of what the mechanical response of periprosthetic bone might be depending on which stem is chosen (Shu et al., 2018). The creation of these “digital twins” would provide a biomechanical basis for choosing a given stem and its length and diameter. Looking at the current literature, the choice at present seems to be at the surgeon’s discretion rather than having any real scientific basis.

Therefore, the objective of this study is to evaluate the biomechanical effect of tibial extension stems on the stability of the tibial component of the TKA, taking into account the mechanical properties of the recipient patient’s bone tissue. This research thus explores the creation of “patient-specific” models that make it possible to determine how stresses will be distributed across the periprosthetic bone and how stable implants will be based on the geometry of the extension stem.

## 2 Materials and methods

### 2.1 Development of the digital model

**FIGURE 1:**
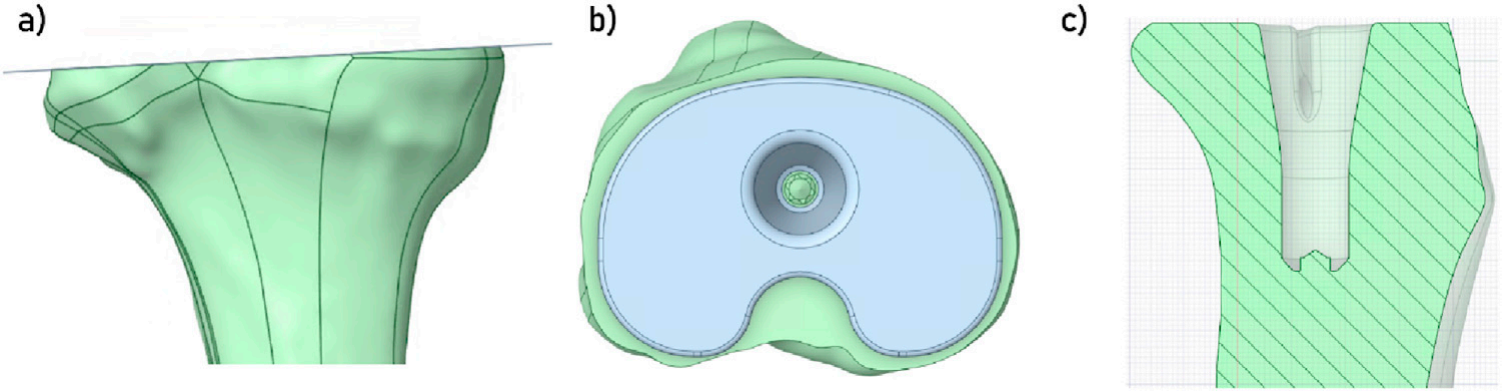
**(a)** Visualisation of the cut plane of the tibial plateau; **(b)** Visualisation of the space occupied by the insert; **(c)** Visualisation of the space prepared for the implant’s insertion in the tibia.

To develop the digital model, CT-scan images of the right knee of an 80-year-old male volunteer suffering from osteoarthritis were used (MR-004, IRB validated). The resulting volumes were composed of voxels of 0.449 × 0.449 × 0.499 mm^3^ in size. Next, the images were processed using semi-automatic volumetric image segmentation techniques using 3D Slicer software (Version 5.6.2, Kitware, France). The 3D geometry of the tibia was extracted and then imported into Ansys SpaceClaim (Version 2024R1, Ansys Inc., United States) to prepare the geometric model. To perform a virtual total arthroplasty, a 2 mm orthogonal cut was made to the tibial epiphysis under the damaged medial plateau at a joint line obliquity (JLO) of 3° (Figure 1a) to model a émechanical alignment of the tibial implant (Rivière et al., 2017). The dimensions of the cementless tibial insert’s endplate (U2 MB, United Orthopedic, Taiwan) were chosen to maximise coverage of the tibial section while ensuring that the implant did not protrude (Figure 1b). The implant positioning was validated by an experienced orthopaedic surgeon.

In this study, we considered press-fit tibial extension stems. Thus, once this implant in position, a boolean geometry operation was performed in the tibia to create a space for the implant (Figure 1c). Several models were developed in order to take into account multiple extension stem dimensions (may it be in diameter or in length). To develop the finite element models, each of the geometric models was then imported into the program Ansys Mechanical (Version 2024R1, Ansys Inc., United States). Discretisation of the different bodies was performed using quadratic tetrahedral elements (TET10).

To define optimal mesh parameters, in order to obtain a good accuracy of the results while maintaining reasonable computation costs, a convergence study was carried out (Ayturk and Puttlitz, 2011). In this study, the element type remained unchanged (TET10), and only the element size was varied. Several models were developed with element sizes ranging from 3 mm to 0.5 mm. An adaptive mesh refinement was applied in the contact region between the tibia and the implant components (tibial baseplate and extension stem), which was the main area of interest in this study. Mesh refinement was continued until the variation in results between two successive mesh densities was less than 5% for key output parameters, including strain, stress, and fracture risk. This ensured that the mesh was sufficiently refined to provide reliable and mesh-independent results in the regions under analysis.

**FIGURE 2:**
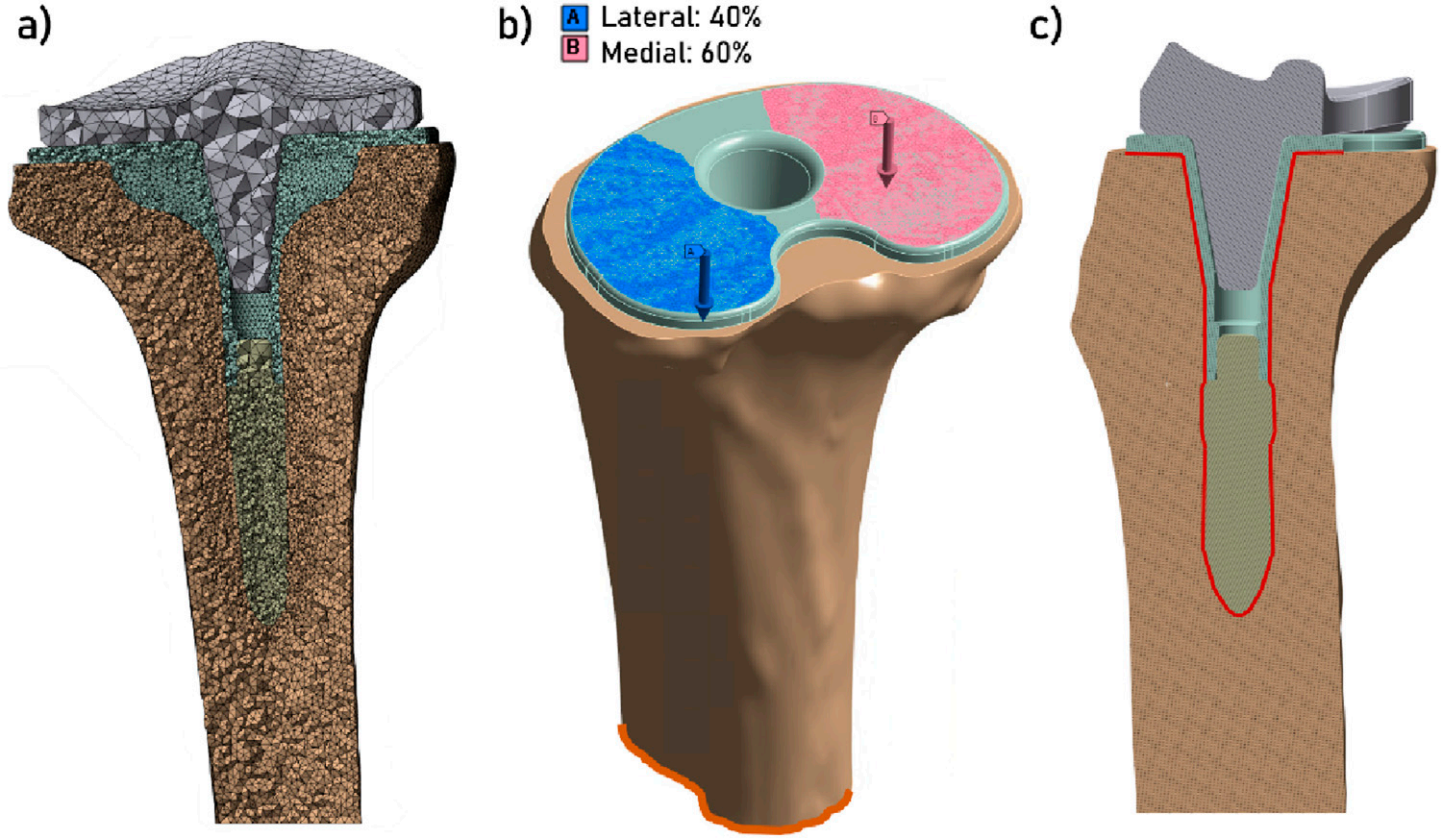
**(a)** Display of the mesh (section view); **(b)** Display of the boundary conditions: increasing force (blue and pink arrows) applied on the superior face of the tibial endplate, with 40% of the load applied on the lateral plateau and 60% of the load applied on the medial plateau (Mündermann et al., 2008; Lee et al., 2025), and the fixed support condition at the distal tibia (in orange); **(c)** Contact zone between the tibia and the implant (section view).

As a result of the mesh convergence analysis, element size for the tibia and the implant was set to 1 mm, whereas in the contact zone around the bone and the prosthesis, element size was set to 0.75 mm (Figure 2a). With these parameters, each model was composed of approximately 2.6 million elements, and could be computed in about 1 hour.

Tibial baseplate and extension stem were assumed to be composed of cobalt–chrome–molybdenum (CoCrMo) (Table 1). For the tibia, the heterogeneous distribution of the patients’ bone density was taken into account (Zannoni et al., 1999; Cattaneo et al., 2001; Schileo et al., 2007), in order to develop patient-specific finite element models. To do that, both the mesh of the tibia and the CT-scan images were imported into a custom-made Python script (QCTMA: pypi. org/project/qctma) used to integrate the values of the CT-scan over each element of the mesh using gaussian quadrature. This protocol is based on the heterogenous distribution of Hounsfield units (HU) in the imaging. A specific density can be associated with each HU (Ese and Waldemar, 2019) and, by the means of a conversion law, can then be linked to a local elasticity modulus. The conversion laws used for both cortical and cancellous bone are shown in Table 1.

**TABLE 1:** Mechanical properties of materials used in the digital models.

Component	Material	Mechanical property	References
Tibia	Cortical bone	$E_{cortical}=3890\times {\rho_{local}}^{2.39}$ ν = 0.3	Snyder and Schneider (1991)
	Cancellous bone	$E_{cancellous}=6570\times {\rho_{local}}^{1.37}$ ν = 0.3	Rho et al. (1995)
Implant	CoCrMo	E = 220 GPa, ν = 0.3	Luyckx et al. (2024)

The density limit for distinguishing between cortical and cancellous bone was set at 1.68 g/cm^3^ (Aubert et al., 2021), as this corresponds to the point where the function defining the modulus of cancellous bone intersects the function defining the modulus of cortical bone.

### 2.2 Structure of the study

In this study, one model was developed for a specific implant configuration, with longer or wider extension stems. A total of six models were developed. Model 1 corresponds to a tibia equipped with an implant used without an extension stem. Models 2 to 6 differ by the dimension of the stem. The dimensions of the extension stems correspond to those proposed in the U2 MB product range. This is summarised in Table 2.

**TABLE 2:** Summary of the implant variations between each model.

	Model 1	Model 2	Model 3	Model 4	Model 5	Model 6
Stem Length (mm)	—	20	45	70	45	45
Stem Diameter (mm)	—	9	9	9	12.5	14

The boundary conditions comprised the fixation of the distal part of the tibia and the application of a compressive force on the superior surface of the implant plateau, with the mechanical axis initially defined at the time of the proximal tibial cut (see Figure 2b). In order to more accurately model the distribution of the force on the physiological tibial plateau, 60% of the load was applied on the medial plateau, and 40% on the lateral plateau (Mündermann et al., 2008; Lee et al., 2025). The rationale for selecting the value of this compression force was that it should represent the stress measured at the tibial plateau during a range of activities, such as: walking in single leg support phase (3 × body weight = 3 × 750 = 2250 N), moving from sitting to standing or going up or down stairs (5 BW = 3750 N), and running (12 BW = 9000 N) (Saxby et al., 2016; Rooney and Derrick, 2013). To represent the case of a secondary stability of the implant (i.e., post osteointegration), a bonded contact condition was imposed between the implant (tibial baseplate and extension stem) and the tibia (Figure 2c).

For each of the six models, strain and stress distribution were observed in the periprosthetic tibial bone, and the minimal principal strain were computed (Wearne et al., 2024). Finally, the risk of fracture of the bone (RF) was computed and presented in percentage. This value is expressed as the ratio between the maximum principal strain in the tibial bone and the ultimate strain limit, which has been described to be equal to 70% of the compressive strain for bone (0.0104), thus equal to 0.0073 (Piovan et al., 2024). These results provide information on the local effects that could result in damage to the bone, potentially damaging implant stability.

## 3 Results

**FIGURE 3:**
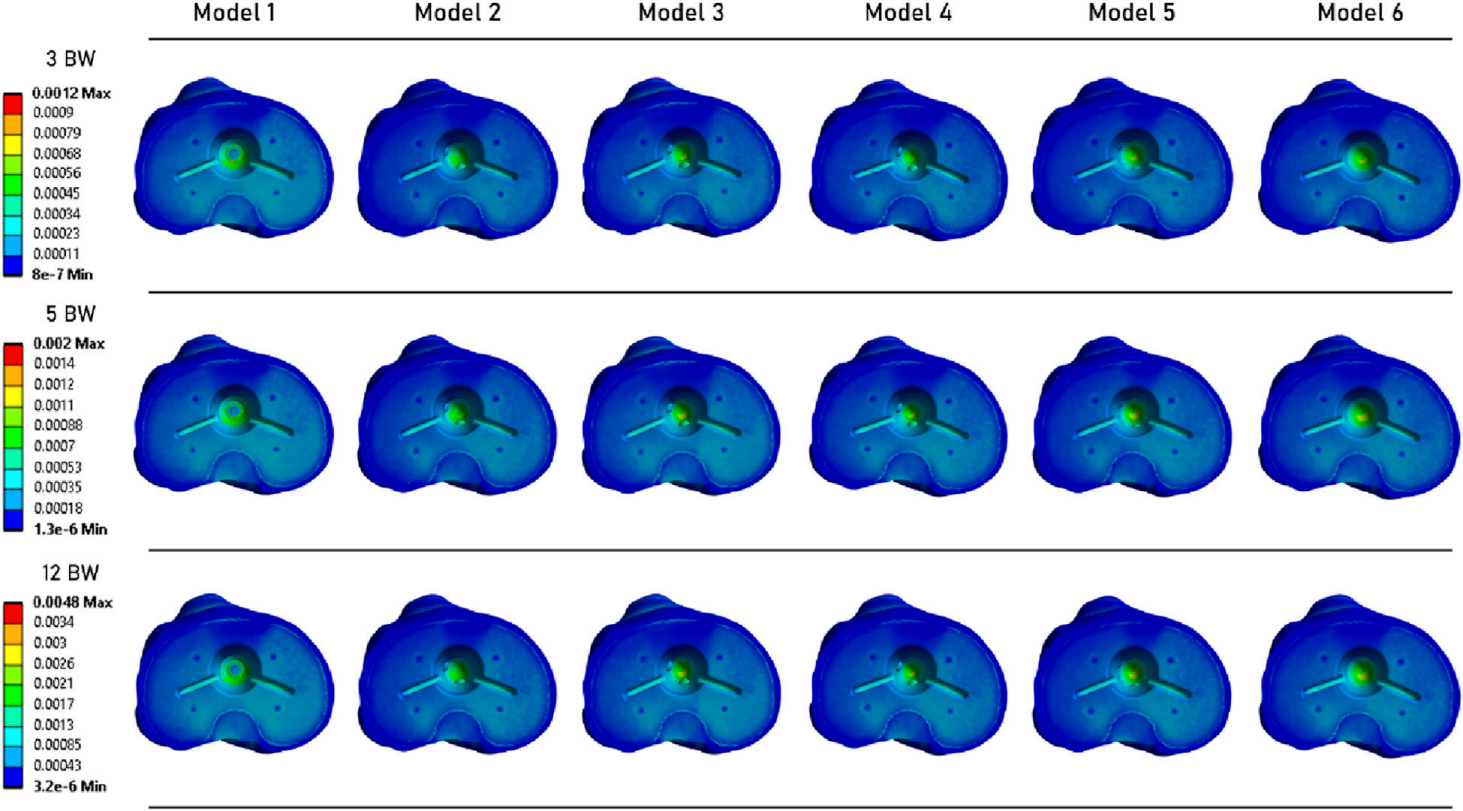
Distribution of strain over the tibial plateau, under the tibial baseplate.

Figure 3 illustrates the distribution of strains in the tibia under the tibial baseplate. The data indicates that models incorporating an extension stem exhibit less strain under the tibial baseplate across all three loading steps. For these models, the strain is primarily located in the bone surrounding the extremity of the stem. However, no difference in strain values was observed in the contact zone where the lateral fins of the implant engage with the tibia.

**FIGURE 4:**
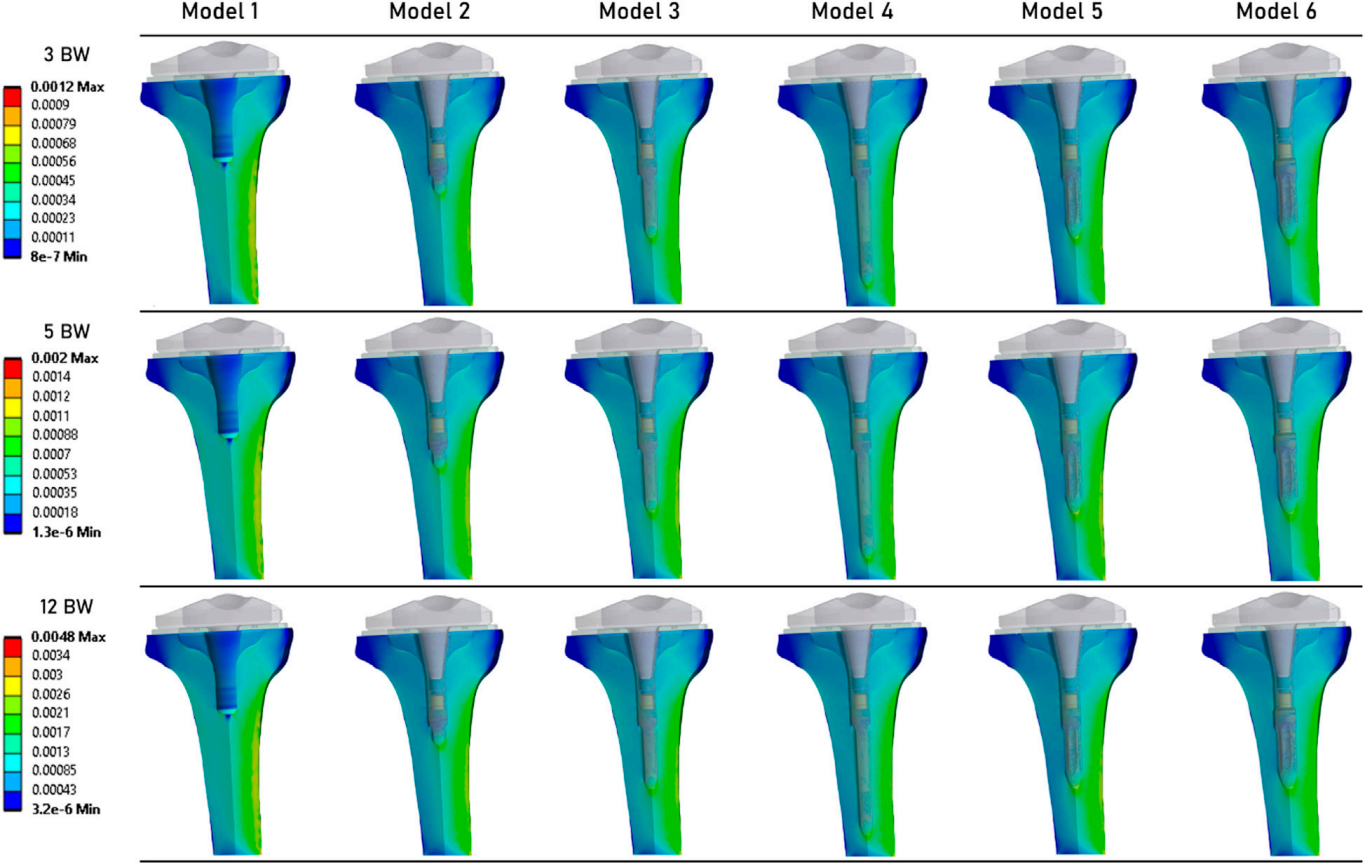
Distribution of strains in the periprosthetic tibial bone.

A similar observation can be made from Figure 4, which shows the strain distribution in the periprosthetic bone. For each loading state, models with a stem exhibit very similar strain distribution. At the 12 BW loading step, Model 1 (without an extension stem) appears to have higher strain levels. However, the highest strain value was computed for Model 5.

**FIGURE 5:**
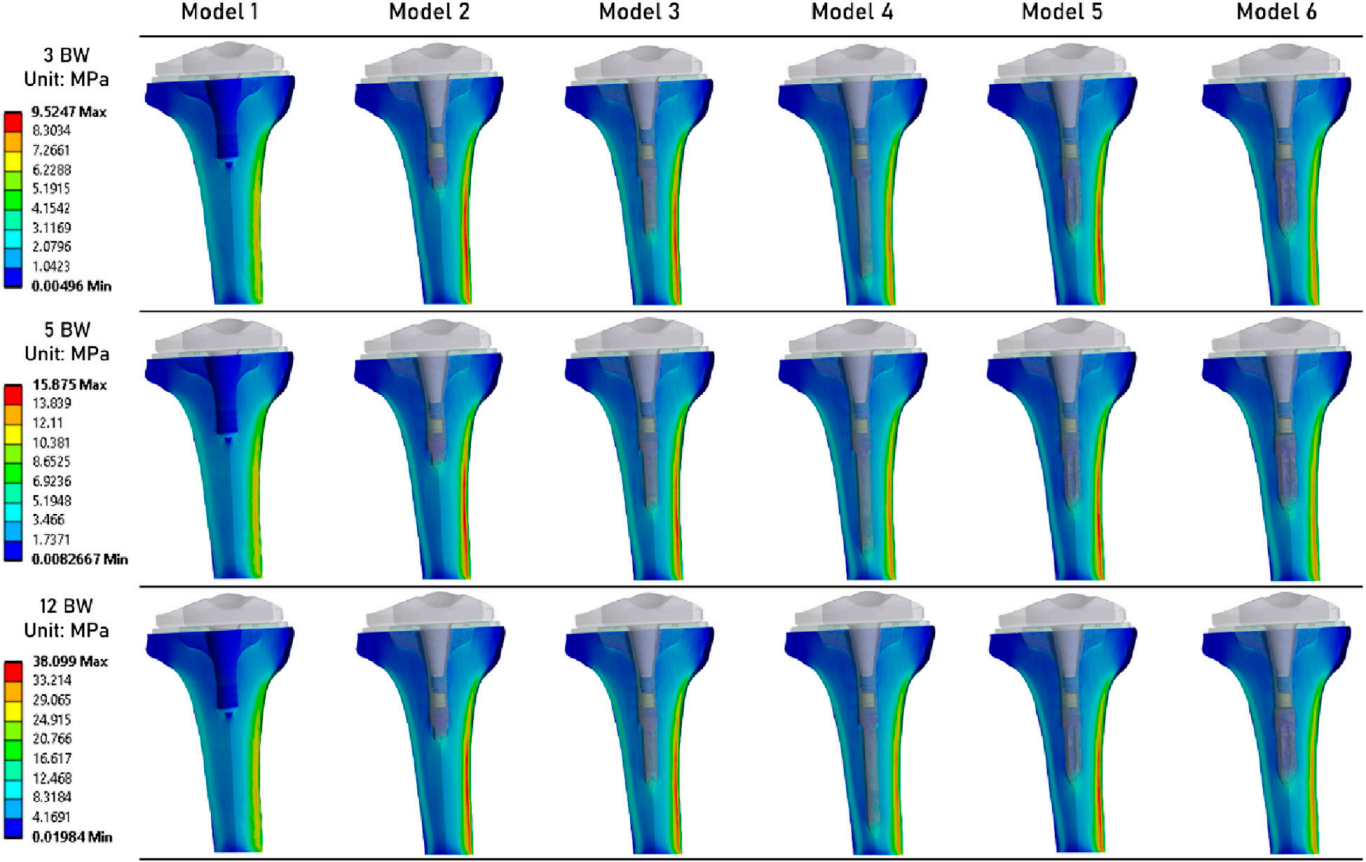
Distribution of von Mises stresses (MPa) in the periprosthetic tibial bone.

Figure 5 shows the stress distribution in the periprosthetic tibial bone. We can observe that although the results are quite similar between all models, the models that implement an extension stem (models 2–6) tend to present higher stress values in the diaphysis cortical bone. The maximum stress values were determined in the Model 5.

**FIGURE 6:**
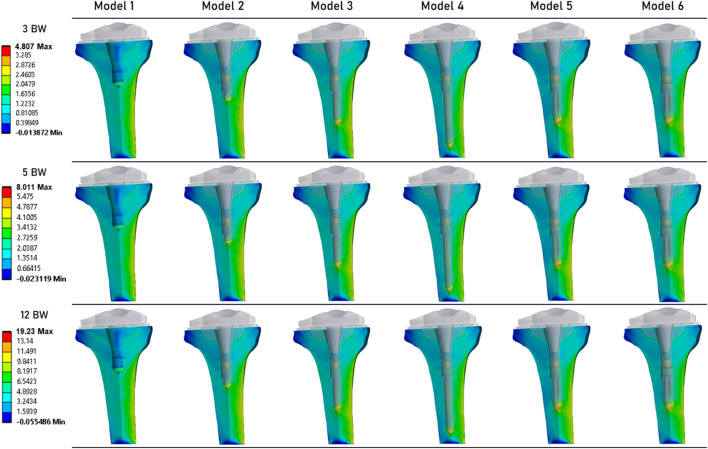
Distribution of the risk of fracture (%) in the periprosthetic tibial bone (Piovan et al., 2024).

Figure 6 shows the distribution of risk of fracture in the tibial bone (Piovan et al., 2024). The most critical zone is located in the bone surrounding the extremity of the extension stem, consistent with the strain distribution shown in Figure 4. The highest fracture risk was observed in Model 5.

Table 3 summarizes the results obtained for each simulation. It presents the maximum values of von Mises stress, equivalent elastic strain, and risk of fracture. For each of these mechanical fields, the lowest values were computed for Model 1. For Models 2 to 6 (which implement an extension stem), von Mises stress values are similar. Specifically, Model 6 (stem of 14 mm in diameter and 45 mm in length) shows values of 8.81 MPa at 3 BW, 14.68 MPa at 5 BW, and 35.24 MPa at 12 BW, compared to Model 2 (stem of 9 mm in diameter and 20 mm in length) with values of 9.52 MPa at 3 BW, 15.88 MPa at 5 BW, and 38.10 MPa at 12 BW. Similar observations can be made for equivalent elastic strain results, with peak strain values of 0.0012 at 3 BW, 0.0020 at 5 BW, and 0.0048 at 12 BW for these models.

**TABLE 3:** Summary of the results of von Mises stress, equivalent elastic strain, risk of fracture, and Minimum principal strain, for each of the 3 loading states.

		Model 1	Model 2	Model 3	Model 4	Model 5	Model 6
3 BW	Von Mises stress (max, MPa)	8.30	9.52	9.37	8.94	9.33	8.81
	Equivalent elastic strain (max)	0.0009	0.0010	0.0011	0.0010	0.0012	0.0010
	Risk of fracture (max, %)	3.29	4.31	4.41	4.28	4.81	4.25
	Minimum principalmicro-strains (median (90th percentile))	−121.56 (−214.67)	−140.59 (−383.67)	−146.01 (−396.69)	−149.37 (−395.17)	−141.74 (−376.05)	−142.55 (−379.75)
5 BW	Von Mises stress (max, MPa)	13.84	15.88	15.62	14.90	15.55	14.68
	Equivalent elastic strain (max)	0.0014	0.0017	0.0018	0.0017	0.0020	0.0017
	Risk of fracture (max, %)	5.48	7.19	7.35	7.14	8.01	7.08
	Minimum principalmicro-strains (median (90th percentile))	−202.60 (−357.78)	−234.32 (−639.45)	−243.36 (−661.15)	−248.96 (−658.62)	−236.23 (−626.76)	−237.58 (−632.92)
12 BW	Von Mises stress (max, MPa)	33.21	38.10	37.48	35.76	37.32	35.24
	Equivalent elastic strain (max)	0.0034	0.0041	0.0044	0.0042	0.0048	0.0041
	Risk of fracture (max, %)	13.14	17.26	17.64	17.13	19.23	16.99
	Minimum principalmicro-strains (median (90th percentile))	−486.25 (−858.68)	−562.36 (−1534.70)	−584.06 (−1586.80)	−597.50 (−1580.70)	−566.95 (−1504.20)	−570.20 (−1519.00)

Finally, we examined the median and 90th percentile values for minimal principal µ-strain, linked to a compressive load (Wearne et al., 2024). These strains were computed only for the mesh elements located in the more refined zone (Figure 1a). We observed that the lowest values were found in the model with the longest extension stem, while the model without a stem produced the higher values.

## 4 Discussion

In this study, various finite element (FE) models of knees with implants were developed to evaluate the biomechanical effects of adding an extension stem on the stability of total knee replacements. These patient-specific models account for the mechanical properties of the recipient’s bone. The results suggest that adding an extension stem tends to reduce strain under the tibial baseplate. However, stress appears to increase in the bone surrounding the extremity of the extension stem, regardless of stem dimensions.

To assess the plausibility of the strain levels predicted by our finite element models, we compared them with published experimental data (Correa et al., 2018). Reported cortical strain magnitudes in the order of a few hundred to about one thousand µ-strain in the tibia under walking and stair-climbing loads, using digital image correlation and strain gauges. The values obtained in our simulations in the cortical bone surrounding the stem tip fall within this range, which supports the realism of our predictions despite the simplifications inherent to the modelling approach.

The geometric parameter of the stem that appears to have the greatest impact on fracture risk is the diameter. Our results show that for each loading state, stems with the largest diameters (12.5 mm and 14 mm) exhibit the largest zones of high fracture risk and strain. A similar observation was previously noted in the literature by Awadalla et al. (2018). This may reflect greater cortical contact with larger diameters under compressive loading.

Our findings align with observations by Completo et al. (2009), who highlighted the significant impact of stem geometry on stress shielding and stress concentrations in the tibial bone. Specifically, they demonstrated that short stems induce less stress shielding and fewer stress concentrations at the stem tip compared to long stems. Additionally, Scott and Biant (2012) noted that although stems improve the mechanical stability of tibial components, they can lead to reduced bone density along their length, consistent with our findings of increased strain at the stem extremity. These insights underscore the importance of optimizing stem design to ensure implant stability while minimizing adverse effects on surrounding bone.

In addition, Eidel et al. (2021) emphasise that optimising force transmission through the tibial plate–replicating pre-surgical loading conditions–can significantly reduce stress shielding. Their work demonstrates that by using a compliant bone-stem interface, achieved through sliding friction conditions, load can be preferentially transmitted through the plate rather than the stem. This is consistent with our results showing reduced strain below the baseplate but increased stress at the stem tip. Designs promoting plate-mediated load transfer may therefore help preserve bone loading and reduce periprosthetic fracture risk. This bionics-inspired strategy offers a promising way to balance implant stability with preservation of bone quality.

These findings should be interpreted as biomechanical tendencies under the bonded interface assumption, and they should not be directly translated into clinical recommendations for press-fit stem selection without additional validation, including explicit modelling of initial stability and micromotion.

In this study, we focused on secondary stability with secure anchorage of the prosthesis in the tibia. Future work could extend this approach to primary stability to assess the impact of anchorage on our results (Eidel et al., 2021). This would also allow for the study of relative displacements between the tibial implant and bone, which were assumed to be negligible in the present work. In addition, the present work did not include a systematic sensitivity analysis of the HU-to-modulus conversion laws or of variations in elastic modulus. Although the chosen density–modulus relationship was consistent with reported tibial bone properties, future studies should test the influence of alternative conversion equations and ±15% changes in modulus to fully quantify their effect on the results. Additionally, our numerical models were based on data from a single patient. These findings should therefore be interpreted as qualitative trends in load redistribution rather than absolute thresholds of clinical risk, since small differences between peak values may not be clinically meaningful and could be influenced by inter-patient variability in bone density and tibial morphology. Developing models from a broader patient base, considering variations in bone geometries and mechanical properties, would help capture this variability more comprehensively. Finally, while we simulated daily activities by varying compressive forces, future studies should also vary the direction of loading (Calliess et al., 2014). This simplification, together with the absence of varus/valgus malalignment, posterior slope variations, and shear or torque components, restricts direct clinical extrapolation of the present findings.

## 5 Conclusion

This study highlights the critical role of tibial extension stems in improving the biomechanical stability of total knee arthroplasty. Our results show that in the case of compressive loadings, the addition of an extension stem tends to increase both stress and strain in the periprosthetic bone, in particular around the extension stem, but seems to decrease their values under the implant’s baseplate. In addition, an increase in stem diameter correlates with a higher risk of fracture due to increased cortical contact within the tibia. Using patient-specific finite element models, this work provides the basis for a surgical planning strategy based on accurate biomechanical data. This approach allows surgeons to optimise implant selection, thereby improving durability and functional outcomes for patients. By integrating these findings into clinical practice, we can potentially improve the longevity of knee replacements and the overall quality of life for patients.

## Data Availability

The datasets presented in this article are not readily available because results obtained from patients imaging. Requests to access the datasets should be directed to MS, mathieu.severyns@hotmail.fr.
